# Detection of small intestine duplication in a 16-year-old girl using small bowel enteroscopy without balloon assistance

**DOI:** 10.1055/a-2503-6137

**Published:** 2025-01-14

**Authors:** Yanqin Long, Haojie Du, Jun Li, Xiaosun Liu, Lihua Chen

**Affiliations:** 1 71069 Gastroenterology, The First Affiliated Hospital of Zhejiang University School of Medicine, Hangzhou, China; 2 71069 Pathology, The First Affiliated Hospital of Zhejiang University School of Medicine, Hangzhou, China; 3 71069 Gastrointestinal Surgery, The First Affiliated Hospital of Zhejiang University School of Medicine, Hangzhou, China

A 16-year-old girl, a high school student, presented with a 2-year history of intermittent pain in the left upper abdomen. Routine laboratory tests performed at the time of admission were normal. Contrast-enhanced abdominal computed tomography revealed an intussusception-like appearance at the duodenojejunal junction ( Fig. 1 ).

**Fig. 1:**
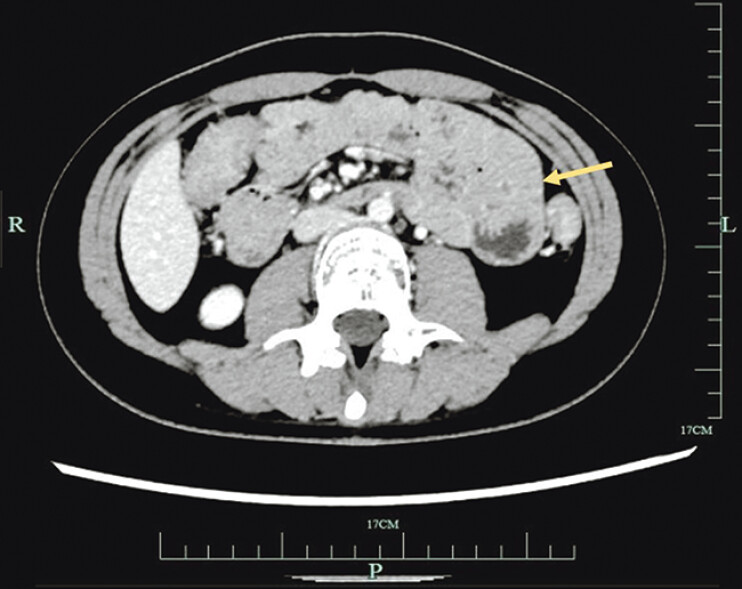
Contrast-enhanced abdominal computed tomography revealing an intussusception-like appearance (arrow) of the duodenojejunal junction.

The patient underwent small bowel enteroscopy without balloon assistance, which revealed a large submucosal lesion at the duodenojejunal junction, measuring approximately 5 × 3 cm. The lesion had a smooth mucosal surface with a depressed area on the oral side ( Fig. 2 , Video 1 ). Surgical resection was subsequently performed. Intraoperatively, a soft, mobile mass located near the ligament of Treitz in the jejunum was excised for pathological examination. Pathological analysis showed ectopic gastric mucosa, including gastric pits and fundic glands, within normal small intestinal mucosa, with mature cellular differentiation. Additionally, a duplicated segment of the digestive tract lined by gastric mucosa and pseudostratified ciliated columnar epithelium, surrounded by a complete muscularis mucosae, was identified ( Fig. 3 ). A final diagnosis of small bowel duplication was established. Postoperatively, the patient experienced no recurrence of abdominal pain.

**Fig. 2:**
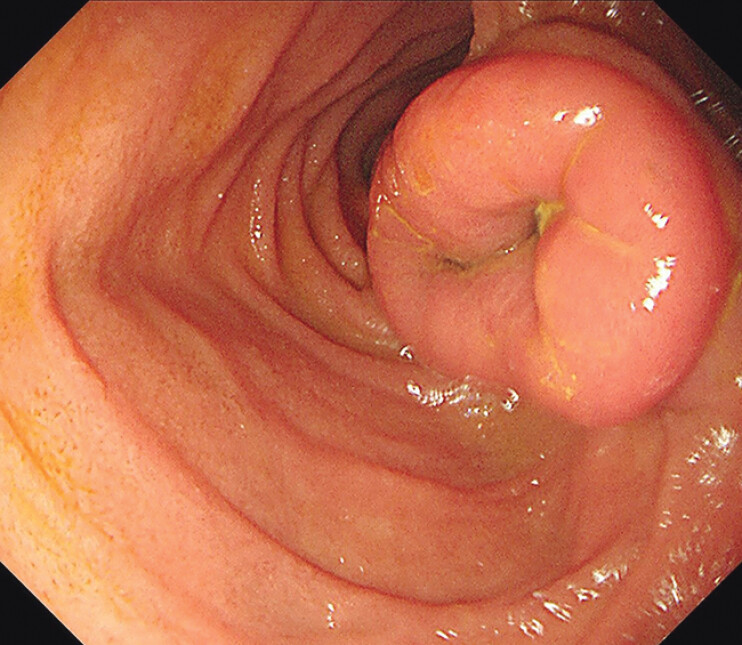
A large submucosal lesion, measuring approximately 5 × 3 cm, located at the duodenojejunal junction is shown, with a smooth mucosal surface and a depressed area on the oral side.

Small intestinal duplication located at the duodenojejunal junction was detected by small bowel enteroscopy without balloon assistance in a 16-year-old girl with intermittent abdominal pain.Video 1

**Fig. 3:**
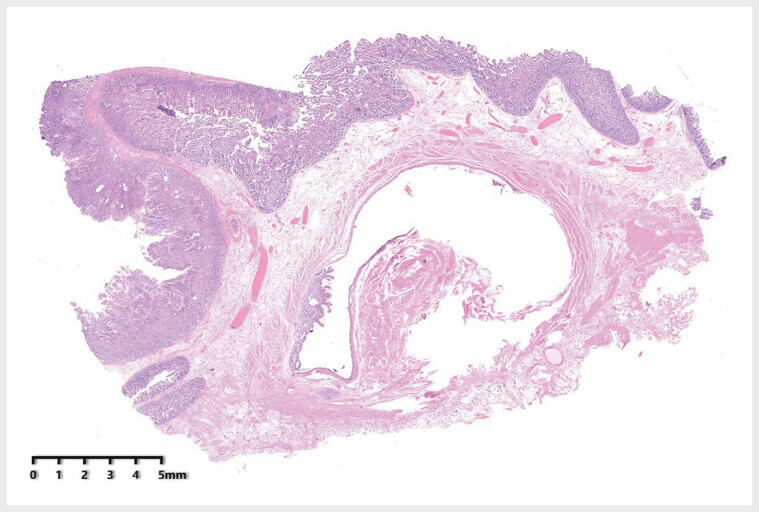
Microscopic examination revealed ectopic gastric mucosa with gastric pits and fundic glands located within normal small intestine mucosa, exhibiting mature cellular differentiation. Additionally, a duplicated segment of the digestive tract lined with gastric mucosa and pseudostratified ciliated columnar epithelium, surrounded by a complete muscularis mucosae, was observed (magnification: 4×).

Gastrointestinal duplications are rare congenital anomalies, usually found in children, with a higher incidence in boys, typically within the first 2 years of life 1 2 . Approximately 2–12% of small bowel duplications occur in the duodenum, 44% in the ileum, and 50% in the jejunum 3 . The clinical presentation depends on the duplication size and epithelial type. Duplications lined with gastric epithelium can secrete acid, leading to ulceration, gastrointestinal bleeding, or acute abdomen in cases of perforation. Other symptoms may include chronic abdominal pain, nausea, vomiting, jaundice, pancreatitis, and an abdominal mass 4 5 . Preoperative diagnosis remains challenging; however, advancements in small bowel enteroscopy allow for precise detection. In this case, enteroscopy provided a clear and comprehensive view of the jejunal lesion. This case will assist endoscopists in promptly diagnosing this condition in the future.

Endoscopy_UCTN_Code_CCL_1AB_2AZ_3AZ

## Notes

Endoscopy E-Videos https://eref.thieme.de/e-videos
*E-Videos* is an open access online section of the journal *Endoscopy* , reporting on interesting cases and new techniques in gastroenterological endoscopy. All papers include a high-quality video and are published with a Creative Commons CC-BY license. Endoscopy E-Videos qualify for HINARI discounts and waivers and eligibility is automatically checked during the submission process. We grant 100% waivers to articles whose corresponding authors are based in Group A countries and 50% waivers to those who are based in Group B countries as classified by Research4Life (see: https://www.research4life.org/access/eligibility/ ). This section has its own submission website at https://mc.manuscriptcentral.com/e-videos .
